# Decoding the Web for Quality Insights on Mouthwash Information in Brazilian Websites

**DOI:** 10.1111/idh.70023

**Published:** 2025-12-04

**Authors:** Bruna Di Profio, Matheus Lotto, Yasmin Teixeira das Graças, Patricia Estefania Ayala Aguirre, Cristina Cunha Villar, Giuseppe Alexandre Romito, Thiago Cruvinel, Cláudio Mendes Pannuti

**Affiliations:** ^1^ Division of Periodontics, Department of Stomatology, School of Dentistry University of São Paulo – FO‐USP São Paulo SP Brazil; ^2^ Department of Pediatric Dentistry, Orthodontics and Public Health, Bauru School of Dentistry University of São Paulo FOB‐USP Bauru SP Brazil; ^3^ Universidad de especialidades Espíritu Santo – UEES Samborondón Ecuador

**Keywords:** consumer health information, COVID‐19, dental informatics, internet use, mouthwashes

## Abstract

**Background:**

The internet is an important source of health information for the population. There is robust evidence about the efficacy of mouthwashes in the prevention and treatment of oral diseases. However, many websites containing mouthwash‐related content may display misinformation and be challenging to read and understand. Thus, this study evaluates the quality of information available about mouthwashes on Brazilian websites.

**Methods:**

A total of 100 websites were evaluated across Google, Bing and Yahoo!. The websites were organised into rankings according to their order of appearance on each of the four search engines. Two independent examiners assessed the quality of the websites using the DISCERN questionnaire and the *Journal of American Medical Association* (JAMA) benchmark criteria. The readability of the sites was assessed by the Flesch Reading Ease adapted to Brazilian Portuguese (FRE‐BP). The content of the websites was categorised according to the presence or absence of information relevant to the theme. Statistical analysis was performed using Spearman rank correlation coefficient, Mann–Whitney *U* test, Kruskal–Wallis test and Dunn post hoc test.

**Results:**

A total of 32 sites were analysed. Web content was considered of poor quality by DISCERN (mean 37.46 ± 8.28) and JAMA (mean 1.37 ± 0.87) scores, presenting difficult reading levels (FRE‐BP: mean 44.04 ± 9.89).

**Conclusions:**

The mouthwash‐related content available on Brazilian websites was considered of low quality and difficult to read.

## 1 Introduction

The internet is an emerging source of health information for the population [1]. A significant portion of the American population turns to the internet for health advice because it is easily accessible, appealing, and often free [2]. The patients' understanding of health and disease conditions enhances their ability to make informed decisions regarding the prevention, diagnosis and management of these conditions [3]. However, most patients lack the technical and scientific knowledge necessary to discern and value reliable information. The abundance of online information has shifted the concern from its availability to the assurance of its credibility, relevance and accuracy [4]. This shift has led to a loss of control over the quality of information accessed [5], with many health‐related websites potentially offering misleading content that is difficult to read and understand [6]. Additionally, the quality of information is further complicated by factors like search engine rankings, website design, and how complex information is presented. All these factors can significantly influence internet searches [7, 8].

Among the various health topics, online interest in oral health information has increased [9, 10, 11, 12, 13, 14, 15, 16], with a particular focus on mouthwashes. Mouthwashes are often indicated as adjuncts to mechanical self‐performed oral hygiene, that is, brushing and mechanical interdental control [17, 18]. Scientific evidence shows that mouthwashes containing antimicrobial ingredients, such as chlorhexidine digluconate, essential oils and cetylpyridinium chloride, offer additional benefits in controlling biofilm and gingivitis [19]. On the other hand, some studies showed the efficacy of fluoride‐containing mouthwashes in preventing root caries [20] and managing dentine hypersensitivity [21]. Despite the clear importance of proper oral hygiene, achieving it remains a challenge for many [19]. Thus, it is essential to understand how individuals take their oral health decisions [22].

Considering the critical role that the quality of online health information plays in patients' decision‐making, this study aimed to evaluate the quality of information available on Brazilian websites regarding mouthwashes.

## 2 Methods

### 2.1 Study Design

This study followed the methodology proposed by Aguirre et al. [9]. The quality of mouthwash information was analysed in Brazilian websites. A specific search strategy was developed and the websites were retrieved through major search engines such as Google Search, Yahoo! and Bing. Duplicates, non‐specific and commercial sites, as well as those that were inaccessible or academically oriented were excluded. The remaining websites underwent a comprehensive evaluation by two independent reviewers using established assessment tools: the DISCERN questionnaire [23], the *Journal of American Medical Association* (JAMA) benchmark criteria [24] and the Flesch Reading Ease adapted to Brazilian Portuguese (FRE‐BP) [25].

### 2.2 Search Strategy

The search strategy was designed from the most relevant terms used on the internet. Initially, mouthwash‐subject in Brazilian Portuguese was entered in Keyword Planner [26] to list the automatic matches available. Their relevance was further analysed on Google Trends, focusing on the variation of their Relative Search Volume (RSV) from 2004 through 2021, including all categories of internet queries in Brazil. After excluding terms that did not have statistically significant search volume, a final search strategy was developed by combining three specific terms (‘enxaguante’ + ‘antisséptico’ + ‘bochecho’), which correspond to the synonyms and types of mouthwash written in Brazilian Portuguese.

### 2.3 Selection of Websites

The websites were selected through the three main search engine tools, Google Search, Yahoo! and Bing according to their market share [27]. The searches were executed on a computer connected to the internet, using browsers with cleared histories and cookies, set up to retrieve only websites published in Brazilian Portuguese and accessed in Brazil.

Subsequently, the websites were registered using Way Back Machine—Internet Archive [28] an online service that archives the information exactly as it was recovered, avoiding changes and updates for future analyses.

The selected websites were dichotomised according to the following categories/topics of information: (I) recommends use of the mouthwashes as an adjunct to mechanical plaque control, (II) raises concerns about alcohol in the composition of the mouthwashes, (III) indicates that mouthwashes should be recommended by professionals, (IV) indicates the presence of fluoride in the composition of the mouthwashes, (V) indicates the presence of chlorhexidine in the composition of the mouthwashes, (VI) recommends mouthwashes for preventing oral diseases, (VII) recommends mouthwashes for preventing and treating periodontal diseases, (VIII) recommends mouthwashes for the treatment of dentine hypersensitivity, (IX) recommends mouthwashes for teeth whitening, (X) recommends mouthwashes for the treatment of bad breath, (XI) recommends homemade recipes for use as mouthwashes, (XII) news published or diffused by mass media, (XIII) recommends mouthwashes for reducing the transmission of Sars‐Cov‐2, and (XIV) clearly states that mouthwashes for preventing or controlling COVID‐19 are controversial.

### 2.4 Assessment of Quality of Websites

The websites were evaluated by two independent examiners (B.D.P. and Y.G.) using the DISCERN questionnaire [23] and the *Journal of American Medical Association* (JAMA) benchmark criteria [24]. The DISCERN questionnaire is commonly applied to assess the quality of written information on health treatment choices. The instrument is divided into the following three sections: (1) reliability of the publication, (2) specific details of the information about treatment choices, and (3) overall quality rating of the document. It consists of 16 questions with a 5‐level Likert scale, where the score ‘1’ indicates that the criterion was not fulfilled and the score ‘5’ indicates that the criterion was completely satisfied. The total DISCERN score varies between 15 and 80, as the second question must be disregarded when the first question is scored ‘1’. Typically, only the results of the first and second sections of this instrument are used to qualify the health content of documents, as follows: very poor (15–26), poor (27–38), fair (39–50), good (51–62) and excellent (63–75) [29].

The JAMA benchmark consists of a series of four qualitative criteria that refer to the description of the authorship (author's name, affiliations and credentials), attribution (effective references of content), currency (presence of dates of posts and updates of information) and disclosure (the statement of any potential conflicts of interest) of websites. For each fulfilled criterion, 1 point is given, with a total score of 0–4.

The websites that were divergently qualified by the examiners were reassessed by the same examiners (B.D.P. and Y.G.) to achieve a consensus.

### 2.5 Readability Measures

The Brazilian Portuguese Flesch Reading Easy (FRE‐BP) [25] was used to assess the readability of the websites based on the following formula: FRE‐BP = 248.835 − (84.6 × syllables per word) − (1.015 × words per sentence). These metrics were calculated using the online tool Legibilidade.com (Legibilidade.com Análise de legibilidade textual, Brazil) [30] through the information of the respective Uniform Resource Locator (URL) of each website. All analyses were performed based on the overall written content downloaded from these links. The reading difficulty of a text is presented according to the following scores: very easy (75–100), easy (50–75), difficult (25–50) and very difficult (0–25).

### 2.6 Statistical Analysis

Data were analysed with the statistical package for Social Science (version 21.0; SPSS, Chicago, USA). The hypothesis of normal distribution of data was not confirmed by the Shapiro–Wilk test. Thus, the statistical analysis to compare dichotomised groups was performed with the non‐parametric Mann–Whitney *U* test. The internal consistency of DISCERN was determined by Cronbach's alpha. The interrater reliability of DISCERN and JAMA scores provided by the independent examiners was assessed by intraclass correlation coefficient (ICC) for the absolute concordance. The correlations between distinct measures were evaluated by the Spearman rank correlation coefficients. *p* values of < 0.05 were considered significant for all analyses.

## 3 Results

### 3.1 Websites

**FIGURE 1:**
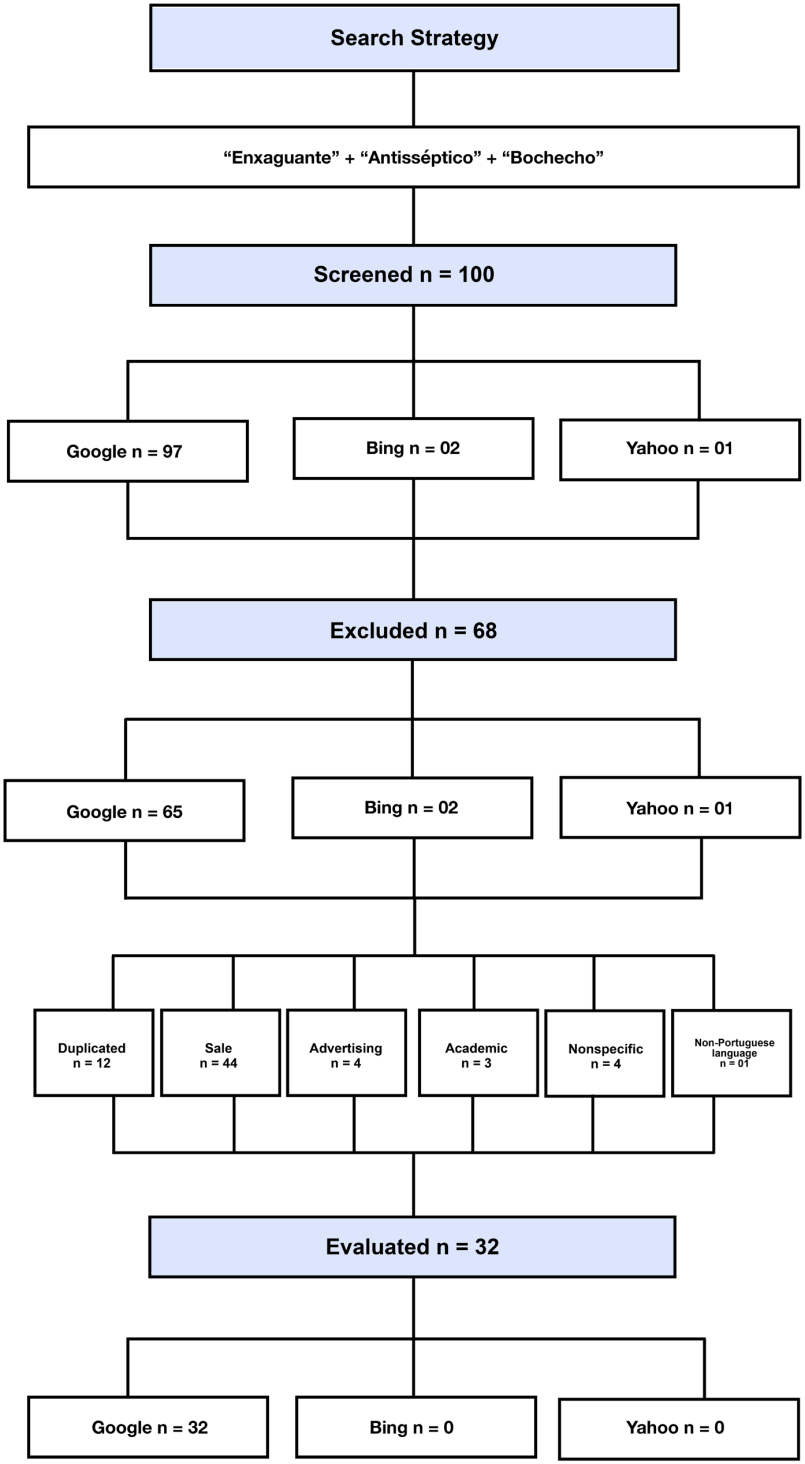
Flowchart depicting the systematic selection of mouthwash‐related Brazilian websites.

One‐hundred websites were retrieved sequentially from Google Search (*n* = 97), Bing (*n* = 2) and Yahoo! (*n* = 1). After applying exclusion criteria for sales‐focused (*n* = 44), duplicated (*n* = 12), advertising (*n* = 4), academic (*n* = 3), non‐specific or inaccessible (*n* = 4) and non‐Portuguese language (*n* = 1) websites, a total of 32 websites were included for evaluation, as depicted in Figure 1.

### 3.2 Internal Consistency and Inter‐Examiner Agreement of Instruments

The assessment of internal consistency and inter‐examiner agreement for the evaluation instruments revealed high reliability. The JAMA benchmark criteria showed an internal consistency of 0.907 and inter‐examiner agreement showed robust values: 0.845 (95% CI 0.483–0.959). The DISCERN questionnaire exhibited even higher internal consistency, with scores of 0.971 for Sections 1 and 2 combined, 0.962 for the total score and 0.802 for Section 3 alone; inter‐examiner agreement showed for DISCERN S1 + S2, 0.933 (95% CI 0.754–0.983) for total DISCERN and 0.690 (95% CI 0.128–0.914) for DISCERN S3.

### 3.3 DISCERN, JAMA and FRE‐BP Scores

The analysis of the DISCERN scores revealed a mean score of 37.46 ± 8.28 with a median of 36, indicating poor‐quality information across the evaluated Brazilian websites regarding mouthwash‐related content (Table 1). Notably, the highest DISCERN score (DISCERN = 66) originated from a mass media website's health information section, whereas the lowest (DISCERN = 23) was associated with an informational website that also sells health products. JAMA benchmark analysis, with a mean of 1.37 ± 0.87 and a median of 1, highlighted that no website achieved the maximum score of 4, and five websites failed to garner any score. According to DISCERN, 6.25% of the websites were rated as very poor, 56.26% as poor, 34.37% as fair, 3.12% as good and none was rated as excellent.

**TABLE 1:** Descriptive statistics of scores of DISCERN, the *Journal of American Medical Association* benchmark (JAMA) and Flesch Reading Ease adapted to Brazilian Portuguese (FRE‐BP).

Outcomes	DISCERN S1^a^	DISCERN S2^a^	DISCERN (S1^a^ + S2^a^)	DISCERN S3^a^	JAMA	FRE‐BP
Mean (SD)	21.03 (4.44)	16.16 (5.13)	37.46 (8.28)	2.21 (1.00)	1.37 (0.87)	44.04 (9.89)
Median	21	14.5	36	2	1	46.32
Minimum	12	9	22	1	0.00	20.32
Maximum	32	27	57	4	3	58.52

*Note:* Significant statistical differences between the groups (Mann–Whitney *U* test, *p* < 0.05).

Abbreviations: FRE‐BP, Flesch Reading Ease adapted to Brazilian Portuguese; JAMA, *Journal of American Medical Association*.

^a^ Three different sections of DISCERN.

JAMA benchmark analyses revealed 46.87% of websites provided authorship information, 15.62% with attribution, 3.12% with currency and 65% with disclosure. Notably, 15.62% of websites did not meet any of the criteria, 40.62% met one, 34.37% met two and 9.37% met three criteria, with none reaching all four criteria for a status of good or excellent. The mean FRE‐BP was 44.04 ± 9.89, with 3.12% being very difficult, 75% difficult, 21.87% easy and none very easy to read.

Analysis of various topics revealed significant differences in information quality between websites (Table 2). Websites emphasising professional prescription of mouthwashes exhibited higher DISCERN scores in sections S1 + S2, S2 and S3. Those recommending mouthwashes for oral disease prevention and dentine hypersensitivity treatment had higher scores in DISCERN section S3. Mass media‐published news demonstrated more accessible reading texts, as did websites recommending mouthwashes for reducing Sars‐Cov‐2 transmission, exhibiting differences in DISCERN section S1. Websites clearly stating the controversy surrounding mouthwash use for COVID‐19 prevention or control achieved better quality and readability scores in DISCERN section S1 (Table 2).

**TABLE 2:** Descriptive statistics of scores of DISCERN^a^, the *Journal of American Medical Association* benchmark (JAMA) and Flesch Reading Ease adapted to Brazilian Portuguese (FRE‐BP).

Categories		Number of sites (total *n* = 32) and percentage (%)	DISCERN S1	DISCERN S2	DISCERN S1 + S2	DISCERN S3	DISCERN	JAMA	FRE‐BP
(I) Recommend use of the mouthwashes as an adjunct to mechanical plaque control	No	*n* = 13 (40.62%)	20.92 (5)	14 (3.85)	35.08 (7.33)	1.85 (0.8)	39 (8.92)	1.38 (1.04)	41.69 (12.24)
	Yes	*n* = 19 (59.37%)	21.11 (4.16)	17.63 (5.45)	39.11 (8.68)	2.47 (1.07)	42 (9.97)	1.37 (0.76)	45.66 (7.85)
(II) Raises concerns about alcohol in the composition of the mouthwashes	No	*n* = 16 (50%)	21.56 (4.17)	16.25 (5.62)	38.12 (8.25)	2.25 (0.93)	42.88 (9.12)	1.56 (0.89)	41.46 (11.74)
	Yes	*n* = 16 (50%)	20.5 (4.77)	16.06 (4.76)	36.81 (8.53)	2.19 (1.1)	39.69 (10.1)	1.19 (0.83)	46.64 (7.06)
(III) Indicates that mouthwashes should be recommended by professionals	No	*n* = 18 (56.25%)	20.78 (4.2)	**13.94 (3.73)**	**34.83 (6.63)**	**1.78 (0.8)**	38.56 (7.73)	1.44 (0.92)	42.79 (11.46)
	Yes	*n* = 14 (43.75%)	21.36 (4.87)	**19 (5.39)**	**40.86 (9.17)**	**2.79 (0.97)**	44.79 (10.87)	1.29 (0.82)	45.66 (7.51)
(IV) Indicates the presence of fluoride in the composition of the mouthwashes	No	*n* = 16 (50%)	20,63 (4.5)	14,44 (4,58)	35,31 (7,56)	2 (0,89)	39,63 (8,41)	1,44 (0,81)	42.34 (11.85)
	Yes	*n* = 16 (50%)	21,44 (4.5)	17,88 (5.2)	39.63 (8.64)	2.44 (1.09)	42.94 (10.68)	1.31 (0.94)	45.76 (7.45)
(V) Indicates the presence of chlorhexidine in the composition of the mouthwashes	No	*n* = 22 (68.75%)	20.73 (4.44)	15.41 (4.87)	36.5 (4.87)	2.14 (0.88)	40.36 (8.31)	1.41 (0.85)	43.18 (10.91)
	Yes	*n* = 10 (31.25%)	21.7 (4.62)	17.8 (5.55)	39.6 (9.64)	2.4 (1.26)	43.3 (12.23)	1.3 (0.94)	45.96 (7.27)
(VI) Recommends mouthwashes for preventing oral diseases	No	*n* = 12 (37.5%)	21.42 (4.29)	14.08 (4.18)	35.75 (7.02)	**1.67 (0.77)**	39.58 (7.74)	1.75 (0.75)	42.62 (10.96)
	Yes	*n* = 20 (62.5%)	20.8 (4.62)	17.4 (5.33)	38.5 (8.97)	**2.55 (0.99)**	42.3 (10.62)	1.15 (0.87)	44.9 (9.37)
(VII) Recommends mouthwashes for preventing and treating periodontal diseases	No	*n* = 14 (43.75%)	21.64 (3.99)	14.43 (4.18)	36.29 (7.17)	2 (0.87)	41.21 (8.1)	1.64 (0.74)	40.98 (11.02)
	Yes	*n* = 19 (56.25%)	20.56 (4.82)	17.5 (5.5)	38.39 (9.15)	2.39 (1.09)	41.33 (10.86)	1.17 (0.92)	46.44 (8.46)
(VIII) Recommends mouthwashes for the treatment of dentine hypersensitivity	No	*n* = 20 (62.5%)	21.2 (4.88)	15.25 (4.64)	36.65 (8.46)	**1.9 (0.96)**	40.5 (10.44)	1.4 (0.88)	42.09 (10.71)
	Yes	*n* = 12 (37.8%)	20.75 (3.79)	17.67 (5.74)	38.83 (8.14)	**2.75 (0.86)**	42.58 (8.28)	1.33 (0.88)	47.32 (7.66)
(IX) Recommends mouthwashes for teeth whitening	No	*n* = 22 (68.75%)	21.64 (4.58)	16 (5.21)	37.91 (8.35)	2.05 (0.95)	41.68 (10.06)	1.41 (0.85)	42.6 (10.68)
	Yes	*n* = 10 (31.25%)	19.7 (4.02)	16.5 (5.21)	36.5 (8.48)	2.6 (1.07)	40.4 (8.94)	1.3 (0.94)	47.23 (7.36)
(X) Recommends mouthwashes for the treatment of bad breath	No	*n* = 13 (40.62%)	21.69 (3.96)	14.92 (3.25)	36.85 (5.74)	2.08 (0.86)	41.38 (6.48)	1.62 (0.76)	42.16 (12.26)
	Yes	*n* = 19 (59.37%)	20.58 (4.79)	17 (6.03)	37.89 (9.78)	2.32 (1.1)	41.43 (11.43)	1.21 (0.91)	45.34 (7.98)
(XI) Recommends homemade recipes for being used as mouthwashes	No	*n* = 31 (96.87%)	21.06 (4.51)	16.35 (5.09)	37.71 (8.3)	2.26 (0.99)	41.61 (9.57)	1.35 (0.87)	43.58 (9.68)
	Yes	*n* = 1 (3.12%)	20	10	30	1	31	2	58.52
(XII) News published or diffused by mass media	No	*n* = 24 (75%)	20.25 (4.5)	16.88 (5.46)	37.46 (9)	2.21 (1.1)	40.71 (10.45)	1.21 (0.83)	**46.69 (7.74)**
	Yes	*n* = 8 (25%)	23.38 (3.54)	14 (3.38)	37.5 (6.14)	2.25 (0.7)	43 (6.69)	1.88 (0.83)	**36.11 (11.83)**
(XIII) Recommends mouthwashes for reducing the transmission of Sars‐Cov‐2	No	*n* = 25 (81.25%)	**20.24 (4.4)**	16.72 (5.4)	37.28 (8.85)	2.24 (1.09)	40.52 (10.28)	1.2 (0.81)	**46.78 (7.59)**
	Yes	*n* = 7 (21.87%)	**23.86 (3.53)**	14.14 (3.62)	38.14 (6.33)	2.14 (0.69)	44 (6.55)	2 (0.81)	**34.28 (11.5)**
(XIV) Clearly states that mouthwashes for preventing or controlling COVID‐19 is controversial	No	*n* = 27 (84,37%)	**20.33 (4.27)**	16.52 (5.24)	37.15 (8.53)	2.22 (1.05)	40.63 (9.98)	1.26 (0.85)	**45.06 (9.62)**
	Yes	*n* = 5 (15.62%)	**24.8 (3.7)**	14.2 (4.43)	39.2 (7.36)	2.2 (0.83)	44.8 (7.01)	2 (0.7)	**38.6 (10.57)**

*Note:* The comparison of means (SD) of quality and readability scores between dichotomised categories of websites. Significant statistical differences between the groups (Mann–Whitney *U* test, *p* < 0.05) are highlighted in bold.

Abbreviations: FRE‐BP, Flesch Reading Ease adapted to Brazilian Portuguese; JAMA, *Journal of American Medical Association*.

^a^ Three different sections of DISCERN.

## 4 Discussion

To our knowledge, this is the first comprehensive evaluation of mouthwash‐related information on Brazilian websites. Unfortunately, our findings indicate widespread poor‐quality content, with many websites that are challenging to read and understand. Our results align with previous investigations on the quality of oral health information online [3, 9, 31, 32, 33, 34, 35, 36].

Remarkably, none of the included websites met all four criteria outlined by the JAMA benchmarks, similarly to that observed in previous studies [3, 37]. The DISCERN analysis revealed variability in the quality of information, ranging from very poor to fair across most evaluated websites. The highest DISCERN score was similar to that found in a study on periodontitis by Kanmaz [3].

A concerning finding from the FRE‐BP analysis, consistent with a previous study [33], indicates that the majority of website content is very difficult or difficult to read. This poses a significant obstacle for individuals with high school education, hampering their comprehension and access to vital information. Notably, the influence of online information on medical consultations is acknowledged, that is, 37.7% of general practitioners and 87.1% of nurses perceive patient‐conducted internet searches as helpful, depending on the accuracy and interpretation of the information [38].

In contrast to established evidence and expert recommendations [18, 39, 40, 41, 42, 43, 44] a substantial number of websites fail to inform the role of mouthwashes as an adjunctive method for effective mechanical biofilm control. Furthermore, there was emphasising lack of emphasis on the importance of professional product recommendations tailored to individual patient needs. However, a majority of the sites do discuss mouthwash use in preventing oral diseases, particularly periodontal disease, in accordance with prevailing evidence [42].

Concerningly, half of the websites omitted the benefits of fluoride in oral health products, despite robust evidence supporting its benefits to dental care [45]. This omission may lead to a lack of awareness about fluoride's role in preventing dental caries, fostering misinformation and potentially increasing the risk of tooth decay.

An intriguing observation is that some websites endorse mouthwashes for dental hypersensitivity without robust evidence [43] and assert whitening effects based on insufficient evidence [44]. Further, misalignment with scientific evidence regarding the purported benefits of mouthwashes in managing halitosis underscores the need for critical evaluation of online health information.

A noteworthy observation is the indication by half of the websites about the dangers of alcohol‐containing mouthwashes, despite a lack of scientific evidence supporting such claims [46]. This highlights a significant issue in online health information dissemination, emphasising the importance of critically evaluating claims to prevent the spread of unwarranted concerns.

Notably, websites suggesting the potential benefits of mouthwash in combating COVID‐19 were primarily from mass media sources, lacking substantial scientific support [47]. Such recommendations may contribute to misinformation, creating a false sense of security among individuals seeking effective preventive measures.

Although dental professionals have occasionally shared misleading oral health information for financial incentives, often promoting alternative or natural treatments [48], the consistently low quality of oral health information online remains a persistent challenge. Internet users face numerous hurdles, including navigating complex websites, information overload, assessing source credibility, dealing with disorganised content and biassed advertising, and deciphering technical jargon that hinders comprehension for less‐educated individuals.

Misinformation or low‐quality information on the internet can have deleterious societal impacts [49]. For instance, the anti‐vax movement, fueled by social media, has caused significant damage in the fight against epidemics, such as COVID‐19. Therefore, future research should identify susceptible populations and understand socio‐demographic and ideological disparities among users [49]. Social media platforms must take responsibility for content moderation, and government regulations should address non‐responsibility for content [50]. Initiatives like Healthy People 2020 prioritise communication in health and the quality of information, acknowledging the potential risk of inaccurate information to population health [51]. However, implementing specific regulations requires careful debate to avoid suppressing freedom of expression and opinion [52].

Our study has limitations, such as the restriction to sites in Brazilian Portuguese, the exclusion of links associated with scientific publications, and the focus on written content. However, the results highlight the urgent need for improved, evidence‐based oral health information on the internet. Further, our findings underscore the urgency of implementing targeted policies aimed at promoting the creation and dissemination of high‐quality information on oral health within the digital space. To make informed decisions about oral health products, individuals should consult dental professionals for personalised recommendations based on their unique oral health needs. Relying on reputable sources, such as scientific studies and dental associations, is crucial when evaluating the safety and effectiveness of oral care products, rather than on claims found on some websites.

## 5 Conclusion

The examination of mouthwash‐related content across a sample of Brazilian websites has revealed consistently low‐quality material that poses challenges in terms of readability.

## 6 Clinical Relevance

### 6.1 Scientific Rationale for Study

The internet is an important source of health information for the population. However, many health‐related websites offer misleading advice to the population.

### 6.2 Principal Findings

Websites that offer information about mouthwashes offer inadequate quality and low readability.

### 6.3 Practical Implications

Websites providing low‐quality information about mouthwashes can pose a threat to patients' oral health decision‐making. Monitoring digital health activities on the internet allows for strategic planning of health literacy initiatives, assessing the quality of information and proactive misinformation management.

## Author Contributions

Bruna Di Profio and Cláudio Mendes Pannuti performed data extraction, analysed the data, prepared figures and/or tables, authored or reviewed drafts of the paper, approved the final draft. Yasmin Teixeira das Graças performed data extraction and analysed the data. Matheus Lotto and Thiago Cruvinel conceived and designed the experiments, performed the experiments, analysed the data, prepared figures and/or tables, authored or reviewed drafts of the paper, approved the final draft. Patricia Estefania Ayala Aguirre, Giuseppe Alexandre Romito and Cristina Cunha Villar authored or reviewed drafts of the paper and approved the final draft.

## Funding

This study was financed in part by the Coordenação de Aperfeiçoamento de Pessoal de Nível Superior—Brasil (CAPES)—Finance Code 001. In addition, this study was financed in part by the grant 2019/27242‐0, Fundação de Amparo à Pesquisa do Estado de São Paulo (FAPESP).

## Conflicts of Interest

The authors declare no conflicts of interest.

## Data Availability

The data that support the findings of this study are openly available in Open Science Framework at https://osf.io/v46kn/?view_only=c1a3813596c94e90a2f77381c7ea8a42.
